# The Ongoing Struggle to Find a Gold Standard for PJI Diagnosis

**DOI:** 10.3390/reports8030155

**Published:** 2025-08-21

**Authors:** Emanuel-Cristian Sandu, Catalin Cirstoiu, Sergiu Iordache, Mihai Aurel Costache, Georgian Longin Iacobescu, Adrian Cursaru

**Affiliations:** 1 Department of Orthopedics and Traumatology, ‘Carol Davila’ University of Medicine and Pharmacy, 020021 Bucharest, Romania; emanuel.sandu@drd.umfcd.ro (E.-C.S.);; 2 Department of Orthopedics and Traumatology, University Emergency Hospital, 050098 Bucharest, Romania

**Keywords:** biomarkers, PJI, periprosthetic, joint infection, diagnosis, gold standard, NGS, culture-negative, AI

## Abstract

Periprosthetic joint infection (PJI) is a devastating complication of joint arthroplasty surgery that is difficult to both diagnose and treat. Misdiagnosing a prosthetic infection has terrible consequences for both the patient and healthcare system. No currently used diagnostic test fulfills the requirements to be considered a gold standard. This shortcoming has been overcome through the implementation of multi-criteria diagnostic protocols elaborated by societies including the Infectious Diseases Society of America, International Consensus Meeting and European Bone and Joint Infection Society, using a combination of clinical, paraclinical and molecular findings in order to achieve the best accuracy in diagnosing PJI. This review aims to survey the current state of the techniques and technologies used for the diagnosis of PJI, investigating the accuracies of serum biomarkers (e.g., C-reactive protein, Interleukin-6, procalcitonin, D-dimers, Serum Intercellular Adhesion Molecule-1), synovial biomarkers (e.g., Antimicrobial peptides, lipocalin-2, leukocyte esterase, calprotectin), tissue biomarkers (e.g., Toll-like receptors, CD15) and advanced molecular techniques (e.g., Polymerase chain reaction, Metagenomic next-generation sequencing), as well as describing their ongoing limitations. In the search for an accurate, inexpensive and fast diagnostic test for PJI, we conclude that the accuracies of the currently studied biomarkers could be further enhanced through the development of novel detection technologies.

## 1. Introduction

Due to the high satisfaction rate of patients undergoing joint replacement surgery and concomitant increase in the availability of implants, we expect that the number of patients with end-stage arthritis seeking this type of treatment will grow exponentially in the near future. A recent report from the OECD indicated average incidences of 172 per 100,000 for hip replacement surgery and 119 per 100,000 for knee replacement surgery, with peaks reaching over 200 per 100,000 in countries such as Switzerland, Germany and Finland for both types of surgery [1]. As with any other major surgery, patients that undergo joint arthroplasty are prone to complications. Even with an incidence as low as 1–2% for primary hip and knee arthroplasties [2], periprosthetic joint infection (PJI) is one of the leading causes of implant failure. The serious nature of this complication is a result of its being difficult to diagnose, the need for multiple surgical interventions and the high financial cost of managing those cases. To put things into perspective, the mean cost for two-stage hip revision for a patient diagnosed with PJI is more than double that of aseptic revision (USD 58,369 vs. USD 22,846), with a similar cost difference in the case of knee revision surgery (USD 56,900 for septic revision versus USD 24,630 for aseptic revision) [3]. Misdiagnosed PJIs can further increase these costs due to additional surgical interventions, lengthy hospital stays and prolonged antibiotic treatments, with the general health and morbidity of the patient also being affected.

We cannot emphasize the importance of correct diagnosis of PJI enough, with its early detection being key for a successful treatment. Even though cases involving virulent bacteria (e.g., *S. Aureus*) will most likely develop clear signs and symptoms of infection, there are cases where the evolution of the infection can be silent; the best example is culture-negative PJI, where the low virulence of the pathogen poses difficulties when using common identification methods [4].

The difficulty of establishing a clear distinction between septic and aseptic degradation of implants, combined with the lack of a gold standard for PJI detection, makes differential diagnosis challenging [5]. In order to overcome this challenge and to standardize the diagnosis, some infection societies have proposed multi-criteria protocols for PJI detection. The similarities and strong points of each definition are listed in Table 1. In particular, we reviewed the guidelines created by the European Bone and Joint Infection Society (EBJIS), the Infectious Diseases Society of America (IDSA) and the Musculoskeletal Infection Society (MSIS), revised in 2018 by the International Consensus Meeting (ICM) [6,7,8].

We can observe that all protocols agree on the consideration of clinical signs with high specificity (e.g., sinus tract or purulence surrounding the prosthesis) as pathognomonic signs of infection, or conclude that two positive bacteriological cultures with the same microorganism are sufficient to establish the diagnosis. In reality, the high specificity of the findings is denied by the low sensitivity, with prosthetic infections having the ability to develop in a subclinical state, making diagnosis difficult [9]. Even though the direct identification of pathogens using bacteriological cultures or more advanced molecular techniques plays an invaluable role in pathological diagnoses [10], the complexity of implant-related infections and the continuous adaptation of bacteria have forced researchers to develop new methods for detection. In this context, novel biomarkers are constantly being evaluated for their utility in PJI diagnosis. Instead of attempting to identify the causative bacteria, they follow a different approach and focus more on detecting the host’s response to the infection.

In this comprehensive review, we aim to highlight the advancements in PJI diagnosis and evaluate different biomarkers and methods that show promise for identifying this infectious complication after joint replacement surgery. The end goal is to find an accurate, inexpensive and fast test that can solely establish the diagnosis, which thus can be promoted as a gold standard.

## 2. Serum Biomarkers

Over the years, the importance of serum biomarkers in the screening and diagnosis of PJI has been proven [11]. The ease of obtaining samples, inexpensive nature of the technique and worldwide availability of the technology are the main advantages of serum biomarkers. However, a minor drawback is represented by the fact that the elevation of such parameters in serum can result from systemic inflammation of non-PJI origin, decreasing the specificity of the tests and their ability to precisely diagnose the disease on their own [12].

### 2.1. Serum CRP and ESR

C-reactive protein (CRP) and erythrocyte sedimentation rate (ESR) are the most extensively used ancillary tests for the diagnosis of PJI [13]. Multiple studies have investigated the accuracy of such biomarkers and have described a wide range of sensitivities and specificities: from 42 to 94% and 33 to 87% for ESR [14] and from 74 to 94% and 20 to 100% for CRP [15], respectively. It is important to mention that many factors can influence the levels of these markers, including systemic inflammatory diseases (rheumatoid arthritis, ankylosing spondylitis), other systemic or local infections, malignancies, preoperative antibiotic administration or low-grade infection with low-virulence pathogens (*Cutibacterium* spp. or coagulase-negative *Staphylococci*) [16,17,18]. Acknowledging this deficiency of the tests, we can better understand the inconsistent accuracy between multiple studies. In a study conducted on patients who underwent hip revision surgery, E. Ghanem et al. found the ideal cut-off point for each parameter to detect PJI, with 30 mm/h showing a sensitivity of 94.3% for ESR and a threshold of 10 mg/L yielding a sensitivity of 91.1% for CRP. Furthermore, combining both ESR and CRP thresholds for PJI diagnosis increased the sensitivity to 97.6% [19]. These values stand as positive indicators for PJI to this day, and elevated CRP or ESR levels are minor criteria included in the 2018 ICM protocol.

### 2.2. Serum Interleukin-6

IL-6 is an anti-inflammatory cytokine that plays a major role in the acute phase of protein production by hepatocytes in the case of infection [20]. Serum CRP levels were elevated 4–6 h after IL-6 stimulation, showing that IL-6 could be an early indicator of immune reactions [21]. Its role in PJI diagnosis has been previously addressed by Berbari E. et al. in a meta-analysis involving multiple serum biomarkers, with IL-6 having the highest pooled sensitivity (0.97) and specificity (0.91) overall [22]. However, a more recent meta-analysis that included more studies and assessed serum Il-6 levels in patients with prosthetic revision surgeries showed a much lower sensitivity (0.72) [23]. A major limitation regarding the use of IL-6 as a standard diagnostic tool for PJI was identified in the case of low-grade infections, especially in shoulder infections known to be caused by less virulent bacteria, in which case the sensitivity of the test was as low as 0.13–0.14 [23]. As it is a non-specific serum indicator of inflammation, IL-6 may yield false positives when trying to detect PJI, for example, in cases of injury, trauma, stress, infection, brain death and other situations [24].

### 2.3. Serum Procalcitonin

The role of procalcitonin (PCT) in sepsis diagnosis is well established [25]. As a marker of infection, studies have tried to assess PCT as a diagnostic indicator of PJI. A recent meta-analysis including nine studies showed that the pooled sensitivity of serum PCT for PJI diagnosis was 0.441, while the pooled specificity was 0.852 [26]. They concluded that the poor accuracy makes PCT unsuitable as a serum marker for PJI diagnosis. PCT is a pro-inflammatory cytokine that is produced in reaction to bacterial endotoxins [27]. As PJI is a local infection that typically develops as a chronic condition [28] with little systemic release of bacterial toxins, we can better understand why prosthetic infection does not consistently trigger elevated PCT levels.

### 2.4. Serum D-Dimers

D-dimers are the result of fibrin degradation under the action of plasmin. It is known that both systemic and local infections promote fibrinolytic activity; thereby, elevated levels of D-dimers may be detected in PJI [29]. A recent systematic review, conducted by Wang R. et al. in 2022, assessed the roles of D-dimers in PJI diagnosis with promising results [30]. The review included 10 studies (1756 participants) and showed combined sensitivity and specificity of 0.81 (95% confidence interval [CI] 0.71–0.88) and 0.74 (95% CI 0.61–0.84), respectively. Their good diagnostic value in detecting PJI is reinforced by the inclusion of D-dimers as a minor criterion in the 2018 ICM definition. Research in this line continues, with further investigations needed to determine thresholds, sampling types and laboratory detection techniques, as well as testing in larger populations.

### 2.5. Serum Intercellular Adhesion Molecule-1

Intercellular adhesion molecule-1 is a cell adhesion molecule that is present in the membrane of various cells, including leukocytes and endothelial cells [31]. Its expression is stimulated by inflammation, and it plays a crucial role in the extravasation of leukocytes at the site of inflammation. After detachment from the cellular wall, it can be detected in plasma as sICAM-1 (soluble ICAM-1). T. Worthington et al. investigated the serum levels of sICAM-1 in patients that underwent hip revision surgery [32]. With a median of 180 ng/mL (range: 104–434) in the group of aseptic loosening compared to 330 ng/mL (range: 136–1050) in the septic group, the results showed a significantly higher serum concentration of sICAM-1 in the latter group (*p* = 0.0002). When using a cut-off value of 250 ng/mL, the test had good accuracy in identifying PJI with a sensitivity of 94% but presented a lower specificity of only 74%. The study concluded that serum sICAM-1 in conjunction with IL-6 could aid in the differential diagnosis of PJI.

## 3. Synovial Biomarkers

In order to complement serum biomarkers, clinicians are now using a broad range of synovial fluid biomarkers to build up diagnostic approaches for PJI with increased accuracy. When analyzing the cellularity of the sampled fluid, a high WBC count or an increase in the percentage of PMNs represent important criteria in current diagnostic protocols, indicating a high probability of PJI. The changes that occur in the synovial fluid during infection have been studied, and multiple biomarkers with excellent diagnostic accuracy have emerged [33]. A different approach to PJI diagnosis, involving local elevation of cytokines targeting the inflammatory process or biomarkers that express direct antimicrobial functions, showed promising results in terms of pathological detection.

### 3.1. Synovial CRP

An increased CRP serum level as a systemic response to infection has been corelated with a concomitant increase in this biomarker in synovial fluid [34]. Parvizi J. et al. investigated 59 samples of synovial fluid collected from patients that underwent total knee arthroplasty revision surgery and compared the accuracy of synovial CRP with the serum biomarker in diagnosing PJI [35]. Using a multiplex ELISA to analyze the samples and a cut-off value of 3.7 mg/L, the test yielded an AUC (95% CI) of 0.91, showing a strong diagnostic potential. In addition, a sensitivity of 84% and specificity of 97.1% were reported—higher than the respective values of 76% and 93.3% obtained with serum CRP. Colin M. Baker et al. strengthened Parvizi’s hypothesis with a recent larger study, in which 621 patients were evaluated [36]. The results were similar to those of the former study, with synovial CRP showing an area under the curve of 0.951 (95% CI), sensitivity of 74.2%, and specificity of 98%. They concluded that synovial CRP has excellent accuracy when used to determine the presence of PJI, with a further improvement of the test being obtained when the values were also correlated with those of serum CRP.

### 3.2. Synovial Antimicrobial Peptides

Antimicrobial peptides (AMPs) are small cationic and amphiphilic peptides that are secreted locally by neutrophils as part of the innate immune response to infection [37]. They have the ability to inhibit bacterial growth by regulating intracellular metabolism, lysis of the cellular membrane and immunomodulation of the host response. Elevated concentrations of AMPs as a direct response to the presence of the bacteria make them perfect candidates for the diagnosis of PJI, yielding higher specificity than other biomarkers that target the inflammation process [38]. In humans, AMPs are present in multiple categories and subcategories; of these, the defensins have been researched most in the context of PJI diagnosis, including α-defensin, β-human defensin 2, β-human defensin 3 and cathelicidin LL-37 [38].

Increased alpha-defensin concentration in the synovial fluid, as detected via lateral flow test or ELISA, is now considered a diagnostic criterion in the 2018 ICM and EBJIS definitions of PJI. The promising results of this biomarker in initial studies, in which a very high sensitivity of 97% and specificity of 97% for PJI diagnosis were reported [39], encouraged others to use it; as such, comprehensive meta-analyses were soon possible. One meta-analysis conducted by Sufian S. Ahmad et al. included 42 articles and noted the pooled sensitivity and specificity of alpha-defensin when using the ELISA technique were still as high as 0.97 (95% CI 0.91–0.99) and 0.97 (95% CI 0.94–0.98), respectively; however, these values were lower when using the Synovasure™ test kit, with a sensitivity of 0.80 (95% CI 0.65–0.89) and a specificity of 0.89 (95% CI 0.76–0.96) [40]. Another systematic review was published by Chen y et al. in December of 2019, who noted similar results favoring the ELISA method for the detection of alpha-defensin in the synovial fluid of patients with PJI [41]. The pooled sensitivity and specificity for ELISA were 92% (86–96%) and 99% (98–100%), higher than the 85% (80–89%) and 96% (94–97%) obtained with the lateral flow test strip. Although there is room for improvement, this simple, prompt and convenient test, in conjunction with other clinical and paraclinical aspects of the patient, can help in accurately diagnosing PJI.

With the success of synovial alpha-defensin in diagnosing PJI, even more antimicrobial peptides have been studied. Synovial fluid samples from 35 patients who underwent joint arthroplasty revision surgery were analyzed using the enzyme-linked immunosorbent assay (ELISA) in order to detect the levels of HBD-2, HBD-3 and cathelicidin LL-37 by H. Gollwitzer et al., revealing significant increases in HBD-3 and LL-37 in the synovial fluid of the patients diagnosed with periprosthetic joint infection when compared with those identified in the samples collected from patients with aseptic loosening [42]. After analyzing the receiver operating characteristic curve, area under the curve (AUC) values of 0.745 for HBD-3 and 0.875 for LL-37 were noted when using these biomarkers for PJI diagnosis, proving their good diagnostic accuracy.

### 3.3. Synovial Lipocalin-2

Lipocalin-2 (LCN2)—also known as neutrophil gelatinase-associated lipocalin (NGAL)—is a glycoprotein which is secreted by neutrophils in the presence of bacteria [43]. It plays an important role in the innate defensive response against bacterial infection due to its ability to disturb iron intake in bacteria. One of the first studies to evaluate the diagnostic ability of synovial lipocalin-2 for PJI was conducted by Deirmengian et al., who investigated 16 synovial biomarkers in 95 patients [33]. LCN2 was one of the biomarkers that detected PJI with a sensitivity and specificity of 100%. However, another study showed lower sensitivity and specificity for this biomarker: only 86.3% and 77.2% [44]. The lower accuracy of the test in the latter study may have been due to variation in the study population and the use of a different detection method. A more recent prospective study, conducted by Huang Z. et al. in 2022, also explored the accuracy of this synovial biomarker when used as a diagnostic tool for PJI [45]. Using ELISA to quantify the levels of LCN2, they showed that the median level of this biomarker in the synovial fluid collected from patients diagnosed with PJI was 135 times higher than that in the aseptic failure group. Furthermore, when setting a cut-off value of 263.5 ng/mL, the test was shown to have excellent diagnostic ability, with an AUC of 0.98, sensitivity of 92%, and specificity of 98%. All of these studies suggest that analyzing the synovial fluid and measuring the levels of lipocalin-2 could facilitate the diagnosis of PJI.

### 3.4. Synovial Leukocyte Esterase

Leukocyte esterase (LE) is an enzyme that is secreted by neutrophils after activation at the site of infection. Detection of LE in urine using a calorimetric strip has been shown to be an excellent test for urinary tract infection [46]. From this first use of LE, its utility has been established in a wider range of diseases, including for the detection of digestive system pathologies [47] and infectious pleural effusion [48]. Its high accuracy in detecting infectious diseases in bodily fluids encouraged J. Parvizi et al. to assess the utility of the LE strip on synovial fluid for the diagnosis of PJI in a first study of its kind [49]. With a sensitivity of 80.6% and a specificity of 100% when only a ++ reading value of the test was considered positive, the detection of PJI using the simple, fast and inexpensive LE strip proved to be a valuable diagnostic tool. The effectiveness of this test was embraced by the International Consensus Meeting later in 2018 and has been considered as an important diagnostic criterion in the proposed protocol since then [8]. When used for the initial screening of PJI, the LE strip test has the potential to shorten the time and lower the costs for an accurate diagnosis [50]. Caution should be exercised when collecting synovial fluid, as contamination with blood could influence the end result of the test. This shortcoming could be overcome through further centrifugation of the sample [50].

### 3.5. Synovial IL-6

As stated before, serum IL-6 showed good accuracy as a diagnostic biomarker for PJI. As part of the inflammatory response to infection, IL-6 is secreted by lymphoid and non-lymphoid cells [24] and, in the case of periprosthetic joint infection, high concentrations of this cytokine have been measured not only in the sera of patients but also locally in the synovial fluid [22]. In order to evaluate the diagnostic accuracy of serum and synovial interleukin-6 in detecting PJI, Li J. et al. reviewed 30 studies as part of a recent comprehensive systematic review [51]. They revealed that synovial IL-6 had higher accuracy in detecting PJI when compared to serum IL-6. Synovial IL-6 showed a pooled sensitivity of 87% (75–93%), specificity of 90% (85–93%), positive and negative likelihood ratios of 8.5 (5.3–13.6) and 0.15 (0.08–0.29), diagnostic odds ratio of 57 (21–156), and AUC of 0.94 (0.92–0.96) [51]. These high values demonstrate the high potential of synovial IL-6 measurement in patients undergoing joint arthroplasty revision surgery in order to determine the nature of the implant failure (i.e., septic or aseptic).

### 3.6. Synovial Calprotectin

Calprotectin is a pro-inflammatory cytokine from the S100 protein family—a group of signaling molecules that plays an important role in innate immunity [52]. Among other roles, calprotectin acts as an endogenous damage-associated molecular pattern (DAMP) molecule and triggers the activation of neutrophils, monocytes, dendritic cells and other leukocytes when it binds to Toll-like receptor 4 (TLR-4) [53] in response to bacterial activity. Numerous studies have been conducted in order to evaluate the utility of calprotectin as a biomarker for inflammation, diagnosis and treatment monitoring in the context of rheumatic diseases [54]. In a more recent prospective cohort study, Warren J. et al. investigated the diagnostic potential of calprotectin for PJI by measuring its concentration in synovial fluid [55]. They included 123 patients who underwent revision for total knee arthroplasty failure. Using a cut-off value of ≥50 mg/L for calprotectin in the synovial fluid, the test showed a sensitivity of 98.1%, a specificity of 95.7%, a positive predictive value of 94.5%, a negative predictive value of 98.5% and an AUC of 0.969 [55]. Considering the excellent diagnostic strength of the test in this study, supported by similar results from previous studies [56], a diagnostic meta-analysis investigating synovial calprotectin for the diagnosis of periprosthetic joint infection was soon published [57]. Peng X. et al. reviewed the existing literature and included a total of seven studies. The pooled sensitivity of synovial calprotectin in diagnosing PJI was 94% (95% CI, 87–98%), the specificity was 93% (95% CI, 87–96%) and the AUC was 0.98 (95% CI 0.96–0.99). Considering the high accuracy of the test, the meta-analysis concluded that synovial calprotectin is a promising biomarker for the diagnosis of PJI. Another important finding of the analysis was that the test expressed a low negative likelihood ratio of only 0.06, making synovial calprotectin an ideal marker to infirm the diagnosis of PJI [57].

## 4. Tissue Biomarkers

Histopathological examination of periprosthetic tissue should be considered a standard procedure in the diagnosis of periprosthetic infections. The histopathological criterion used to establish the differential diagnosis is the presence or absence of polymorphonuclear neutrophils in the examined tissues. During histopathological examination of the periprosthetic tissue, the presence of at least five neutrophils per high-power field in five high-power fields at a magnification of 400× is indicative of periprosthetic infection [58]. Some authors have also investigated the presence of other cells, such as lymphocytes or plasma cells [59]. Although PMNs are found in infected tissues, their presence in healthy tissue is minimal or absent. According to all diagnostic tests, if we increase the limit at which the histological test becomes positive from 5 neutrophils to 10 neutrophils, we reduce the sensitivity and increase the specificity; if we decrease it to 1 neutrophil, the reverse situation applies. The MSIS proposed a value of 5 neutrophils as it is the most commonly used value worldwide, and several studies have shown that there are no major differences between 5 and 10 neutrophils [60]. However, certain microorganisms—especially coagulase-negative *Staphylococci* (ConS) and *P. acnes*—can cause periprosthetic infections with a PMN infiltrate of less than five neutrophils per examined field [61].

Morawietz reached a consensus that led to the emergence of a histopathological classification of peri-implant tissues [62], dividing them into four major categories:

1. Type I—particle disease;
2. Type II—infectious tissue (presence of a large number of neutrophils);
3. Type III—mixed tissue (I and II);
4. Type IV—indeterminate tissue—predominantly connective—fibrous.

Bori G. et al. attempted to summarize the utility of histopathologic examination for the diagnosis of PJI in a review where they investigated more than 30 studies [63]. The pooled accuracy of the test was hard to assess as various authors used different cut-off points (from 1 to 10 PMNs per high-power magnification field), evaluated more than one particular joint (knee, hip, shoulder) and collected different tissues (synovial tissue, interface membrane, periprosthetic tissue, joint pseudocapsule). The sensitivity of the test varied from 18% (only one study) to 100% and the specificity from 64% (excluding one study, in which fractures were assessed and 55% specificity was noted) to 100%; however, it should be noted that more than two-thirds of the studies reported a sensitivity over 80% and a specificity over 90% for the test.

### 4.1. CD15

Most studies conducted on this topic have used the hematoxylin and eosin staining technique in order to quantify the presence of polymorphonuclear neutrophils in the periprosthetic tissue [64]. The identification of neutrophils can sometimes be difficult, even if the examiner uses the protocol proposed by Feldman et al. [65]. A possible strategy that could favor the development of morpho-molecular histopathological diagnostic approaches is the use of molecular markers contained in PMNs. Two authors have used this approach in recent clinical studies, but with different methods. In 2009, Morawietz et al. [66] used immunohistochemistry (CD15 antibody) while, in 2015, Kashima et al. used only histochemistry [67]. CD15 is a transmembrane glycan that is expressed on the surface of neutrophils, which plays important roles in the phagocytosis process [68]. It can act as an epitope for a specific CD15 antibody in the immunohistochemistry test and, thus, can be used to identify neutrophils in the examined tissue. The study conducted by Morawietz et al. concluded that 23 PMNs in 10 HPFs (high-power fields) was the limit to establish the differential diagnosis between septic and aseptic degradation of implants. In this study, in contrast to previous clinical studies, the identification of PMNs was not based only on cell morphology but also on immunohistochemistry. Ideally, PMNs can be identified by small lobulated nuclei and a narrow cytoplasmic ring; however, the prosthetic wear particles and bone fragments that are often found in the periprosthetic membrane can make precise sectioning of tissue difficult, leading to artifacts or sections that are too thick and complicating the precise identification of PMNs. The authors concluded that immunohistochemistry increases the accuracy of PMN detection compared to standard stains. Using 23 PMNs on 10 HPFs as a threshold, the histopathological examination had a sensitivity of 73% and specificity of 95% when compared with microbiological diagnosis (AUC of 0.881), and a sensitivity of 77% and specificity of 97% when compared with clinical diagnosis (AUC of 0.891) [66].

Kashima et al. [67] reported that more than two PMNs per microscopic field showed higher sensitivity and accuracy in diagnosing infection. In this study, the authors used a histochemical method using the enzyme chloroacetate esterase (CAE) to identify PMNs. Similarly to the previously presented study, the use of this reaction increased the sensitivity of the histopathological test. Both authors reached a similar conclusion; namely, 23 PMNs in 10 high-power microscopic fields or at least 2 PMNs per field are required to establish the diagnosis. In relation to the initial histopathological diagnostic limit of five PMNs per field proposed by MSIS for the diagnosis of periprosthetic infections, the value of five PMNs was considered to be too high by the aforementioned authors.

### 4.2. Tissue AMPs

Due to the resounding success obtained with the increased concentrations of alpha-defensin and other antimicrobial peptides detected in the synovial fluid of patients with prosthetic infections, few researchers have tried to study their presence in periprosthetic tissues. The presence of HBD-3 and LL37 in the synovial membrane of patients with PJI was first described in a recent study conducted by Banke et al. in 2020 [69]. They addressed a particular case of PJI in which diagnosis is very difficult due to a low-grade infection; specifically, patients with coagulase-negative *Staphylococci* were included. After an immunohistochemical analysis of tissues collected from patients with aseptic loosening and PJI, they found elevated levels of HBD-3 and LL-37 peptides (up to 20× higher) in the latter group. Furthermore, the area under the curve was equal to 1.0 for both biomarkers, revealing excellent diagnostic accuracy. The relevance of this finding can be seen mainly for patients in which synovial fluid is not available or insufficient for sampling.

### 4.3. TLR 1,6

Another less-studied marker for PJI diagnosis are Toll-like receptors (TLRs)—transmembrane receptors that recognize pathogen-associated molecular patterns (PAMPs) and play important roles in activating the inflammatory cascade against bacterial infection [70]. Some TLRs, such as TLR-1 and TLR-6, are triggered by bacterial lipoproteins, making them good candidates as effective biomarkers for the diagnosis of PJI [71]. A study investigating 59 patients who underwent revision of hip or knee arthroplasty concluded that the expression levels of TLR-1 and TLR-6 receptors were significantly higher in the periprosthetic tissues collected from patients with prosthetic infections compared to those with aseptic degradation [72]. Both receptors showed high specificity and sensitivity for the diagnosis of PJI, with TLR-1 outperforming TLR-6, with a specificity of 100% and a sensitivity of 95%. The main disadvantage of this technique is the time required to process periprosthetic tissues for RNA extraction and PCR (polymerase chain reaction) analysis.

## 5. Bacterial Identification

Identification of the causative microorganism is essential for the diagnosis and treatment of PJI. Considering that PJI is an implant-related infection, bacteria that evolve on the surface of the prosthesis in a unique biotic community called a biofilm express a different phenotype than standard planktonic bacteria [73]. Biofilm bacteria can exist in a more latent state with slow metabolism, low virulence and a very slow growth rate. In the presence of biofilm bacteria, existing detection methods may face difficulties in identifying the pathogen.

### 5.1. Bacteriological Cultures

Standard microbiological culture is one of the most-used techniques worldwide for bacterial identification. Considering that this method was first implemented to diagnose acute infections caused by free bacteria, it has some shortcomings when used to identify biofilm bacteria. Due to the slow metabolism of biofilm bacteria, the current recommendation is to culture inoculated samples for at least 14 days before reading the results [74]. Kheir M.M. concluded that collecting a minimum of five intraoperative samples and waiting for a minimum of 8 days before considering a negative result of the cultures should increase the sensitivity of the test [75]. Another particularity of the PJI pathology is represented by the strong binding of the bacteria onto the surface of the implants. An inability to detach the biofilm from the prosthesis affects the low sensitivity of this method [76]. This issue can be overcome by sonication of the implants followed by the inoculation of cultures with the obtained sample. This process has been shown to increase the accuracy of the test in a recent systematic review conducted by Watanabe S. et al., where the cultures from the sonication fluid achieved the greatest accuracy [77]. After analyzing 32 studies, the pooled accuracies of the tests used to diagnose PJI showed a sensitivity of 63% (95% confidence interval, 56% to 70%) and a specificity of 96% (95% CI, 93% to 98%) for the preoperative fluid culture, a sensitivity of 71% (95% CI, 63% to 79%) and a specificity of 92% (95% CI 86% to 96%) for the intraoperative tissue culture, and a sensitivity of 78% (95% CI, 68% to 85%) and a specificity of 91% (95% CI, 83% to 95%) for the sonication fluid culture, proving the superiority of the latter in identifying the causative bacteria [77]. In order to achieve the best results—when the condition of the patient allows—clinicians should always discontinue antibiotics for at least 14 days before collecting samples [78].

### 5.2. Molecular Techniques—PCR, MALDI-TOF/MS, mNGS

Acknowledging the limitations of bacteriological cultures in diagnosing PJI and considering the seriousness of the pathology, the probability of false negative results should not be neglected. When the patient presents clear signs of infection or the suspicion of infection is very high but cultures fail to identify the causative bacteria, a particular case of PJI should be considered. Culture-negative PJIs are reported to have an incidence of 5 to 40% and consume significant resources [79]. Facing this type of pathology more often, researchers in the field of clinical microbiology have had to look for more advanced and accurate techniques, and, for the past decade, their efforts have primarily been concentrated on developing molecular-based diagnostic methods.

PCR (polymerase chain reaction) targeting the 16 s ribosomal region has been successfully used to detect bacteria in PJI patients [80]. Furthermore, this method has evolved into more sensitive variants, in the form of targeted PCR (to detect a specific bacterial species) or multiplex targeted PCR (targeting the most common bacteria associated with prosthetic infection). The diagnostic potential for PJI using this method has been investigated in a meta-analysis published by Qu X. et al. [81]. After investigating 14 studies, they showed a pooled sensitivity of 86% and a specificity of 91% in identifying the infection, thus promoting this technique as an important diagnostic tool (especially in the cases of culture-negative PJIs).

Another molecular technique is shotgun metagenomic next-generation sequencing (mNGS), which targets all the nucleic acid in the probe followed by DNA sequencing in order to identify all microorganisms. Its utility for PJI diagnosis has been evaluated in a systematic review conducted by Tang Y et al., where a sensitivity ranging from 63% to 96% and specificity of 73% to 100% were noted [82]. Another important finding of the study is that this technique can optimize the diagnosis for culture-negative PJI patients or patients with antibiotic administration prior to sampling. When compared to PCR-based molecular diagnostic techniques, NGS presents certain advantages such as a wider range of pathogens that can be identified, improved sensitivity, increased efficiency and the capability to handle high workloads [79]. At present, the widespread application of NGS regarding this pathology is hindered by the limitations of the technique, such as the possibility of false positive results due to contamination or commensal flora, lack of standardization between laboratories, long processing times and high costs [83].

MALDI-TOF/MS (Matrix-Assisted Laser Desorption Ionization—Time of Flight/Mass Spectrometry) is routinely used for the identification of bacterial species from isolated colonies obtained from cultures. This technique allows for the identification of bacteria based on the specific mass/charge ratio of ionized peptides or particles from the bacterial wall surface after they are exposed to a soft laser [84]. The application of this technique was further investigated in a novel study conducted by Beguiristain et al., who investigated 107 arthroplasty revision cases and attempted to diagnose PJI when using MALDI-TOF/MS directly on the collected samples [85]. MALDI-TOF/MS managed to successfully identify 69% of PJI cases with a specificity of 94% from the blood culture bottles inoculated with sonication fluid. While not being overly sensitive, the method still showed an improvement over the classic cultures that yield a sensitivity of only 53%. In another study published by Kuo et al., who used the same technique on synovial fluid inoculated in blood culture bottles obtained from 77 patients, the authors managed to distinguish a sensitivity of 80% for the test [86]. Both studies highlighted two advantages of this technique. When we remove a subculture step and directly use MALDI-TOF/MS on the collected samples, the time required to obtain the final results is more than three times shorter [85,86]. To gain this advantage, direct MALDI-TOF/MS sacrifices sensitivity and the ability to identify polymicrobial infections or fungi when compared to routine MALDI-TOF/MS. The second important asset of this technique is its incredibly low cost (USD 0.50 per specimen) and ease of use; furthermore, the technology is already available in most clinical laboratories [87]. Considering these advantages, combined with the relatively good accuracy of the test noted in these initial studies, the technology should be further evaluated for its utility in the diagnosis of periprosthetic joint infection.

### 5.3. Microcalorimetry

Isothermal microcalorimetry (IMC) is a technique used to identify bacteria based on the heat produced by microbial growth. The main advantage of this method is the ability to detect bacteria much faster than when performing standard microbiological cultures [88]. This fast and accurate detection technique has proven its utility in orthopedic infectious pathologies, successfully diagnosing septic arthritis from synovial fluid samples with a sensitivity of up to 89% and a specificity of 99% [89]. The research continued, and IMC was further evaluated for the detection of bacteria in patients with PJI. A study that included 107 patients with septic and aseptic failure of the prosthesis compared the accuracies of synovial fluid culture and microcalorimetry for PJI diagnosis [90]. Morgenstern C. et al. noted that synovial fluid culture and microcalorimetry had the same sensitivity of 39%, and the results were concordant in 92% of the cases. While the sensitivity of both tests was low, the study emphasized the major difference in time required to obtain positive results. The median time to positivity of IMC was only 9 h (range 1–64 h), compared to 3 days for cultures (range 1–14 days) [90]. Another recent study conducted by Cichos KH et al. explored microcalorimetry as a diagnostic test for PJI, comparing the obtained results with those from standard cultures inoculated with intraoperative deep tissue samples [91]. IMC showed a sensitivity of 83% compared with a sensitivity of only 69% in the case of standard cultures, with both tests having a specificity of 100%. Similarly to the previous study, a key finding was the difference in the median time to detection between the two identification methods, with IMC improving this parameter by almost 2 days [91]. Considering the rapid availability of the results in conjunction with the superior accuracy of the test, as observed in the latter study above, isothermal microcalorimetry definitely has its place in the PJI detection process; however, further studies are required in order to fully assess its diagnostic value.

## 6. Discussion

Periprosthetic joint infection is a catastrophic complication that typically requires multiple surgical interventions, longer hospital stays, prolonged antibiotic treatment and is associated with a poorer functional outcome when compared with aseptic loosening [5]. The inconsistent clinical manifestations and paraclinical findings of the disease make its diagnosis extremely difficult. A fast preoperative diagnostic is key for the successful treatment of this condition, considering that septic and aseptic loosening have completely different therapeutic routes. After reviewing the most recent publications regarding this pathology, a gold standard for the diagnosis of PJI is still unavailable, with recently investigated tests showing insufficient accuracy to solely diagnose the infection. As presented at the beginning of this review, multiple infection societies have attempted to overcome this challenge by implementing diagnostic protocols including multiple criteria. Comparing the accuracies of the protocols when addressing the preoperative diagnosis, the diagnostic algorithm proposed by EBJIS showed the highest sensitivity of 89% and a negative predictive value (NVP) of 90%, the ICM protocol was second with a sensitivity of 85% and an NVP of 87% and the IDSA protocol showed a sensitivity of only 56% and NVP of 77% [92]. At present, diagnosis is established using various serological and synovial biomarkers, clinical signs, histological and microbiological tests, intraoperative findings and more complex molecular techniques [8]. The available literature data evaluated while elaborating this review presented a certain degree of consistency, as can be observed in Table 2. Furthermore, we observed that biomarkers such as alpha-defensin, leukocyte esterase and histopathological findings, which are already used as diagnostic tools by the aforementioned societies, showed high accuracies.

The most commonly used serum biomarkers for PJI screening are CRP and VSH. The most appealing advantages of these markers include the ease of collecting samples, low cost and fast results. Clinicians should understand that these comprise only an ancillary test for PJI diagnosis and do not possess the accuracy to rule out an infection [16]. A low-grade infection can evolve with normal serum findings; on the other hand, the levels of these biomarkers may also be elevated in the presence of other systemic inflammatory diseases (e.g., rheumatological diseases, other infections). The accuracy of novel serum biomarkers such as sICAM, IL-6 and D-dimers were evaluated in this review in terms of their diagnostic potential regarding PJI, showing good results [22,30,32].

Considering that PJI is a local infection, clinicians and researchers have assessed synovial biomarkers in order to increase the accuracy of the aforementioned tests and improve the diagnostic process. Analyzing the synovial fluid concentrations of lipocalin-2, leukocyte esterase, IL-6, calprotectin and AMPs revealed some of the highest sensitivities and specificities in diagnosing PJI [33,40,49,51,57]. In order to minimize the risk of contamination, joint aspiration should be performed using a sterile technique and with a needle of adequate length (e.g., some overweight patients may require the use of a spinal needle). Joint aspiration can also be performed during surgery. Collecting the synovial fluid after incision of the skin, piercing only the joint capsule, further increases the accuracy of the microbiological test [93]. For the hip joint, clinicians can perform aspiration under X-ray or ultrasound guidance, with the latter method more often facilitating successful sampling [94]. The clinician should plan which tests will be performed on the acquired synovial fluid in order to ensure that an appropriate volume is aspirated. The amount of fluid required for the most commonly used tests ranges from 20 µL to 2 mL; for example, 0.5 mL is sufficient for alpha-defensin and cell count testing, 2 mL is sufficient for NGS and a minimum of 1 mL is sufficient for bacteriological cultures (with slow-growing bacteria requiring higher volumes) [95].

Researchers studying infectious diseases have recently shown increased interest in peptides with antimicrobial properties and their roles in the pathology and diagnosis of PJI. With the detection of increased concentrations of alpha-defensin in the synovial fluid of patients with PJI already serving as an important criterion in the ICM protocol [8], AMP testing could be considered a promising candidate for gold standard status in the future. High levels of other defensins (e.g., HBD-2, HBD-3 or cathelicidin LL-37) have been identified not only in the synovial fluid from patients with PJI but also in periprosthetic tissues via immunohistochemistry, enhancing the standard histopathological examination [69].

While providing excellent results for the diagnosis of PJI, routine alpha-defensin testing is hindered by the high cost of lateral flow testing and the need for a specialized laboratory and personnel for ELISA testing. The use of proteomics technologies for the diagnosis of PJI has been highlighted by Iorio et al. in 2021, who successfully managed to diagnose the disease by detecting high levels of alpha-defensin in synovial fluid using MALDI-TOF/MS [96]. The overall accuracy of the test was 94.9%, with a sensitivity as high as 93% and a specificity of 96%. Considering that this technology is already widespread, has an incredibly low cost (EUR 0.73 per sample vs. USD 125 for immunoassay) and requires a short time to obtain results (20 min), MALDI-TOF/MS could be a promising alternative for synovial alpha-defensin testing in order to accurately diagnose PJI.

Taking into account the low sensitivity of microbiological cultures—especially when assessing less-virulent biofilm bacteria—the development of more advanced molecular techniques for bacterial identification, such as PCR and mNGS, has majorly impacted the overall management of PJI [97]. While these novel methods can be expensive to implement and perform, require qualified staff and are prone to false positive results at present (taking into account the overly sensitive nature of the tests), they remain the last option when facing culture-negative PJIs [82]. Recent advancements in artificial intelligence (AI) and machine learning (ML) can aid researchers in processing the large amount of data obtained via genetic sequencing, particularly in terms of extracting meaningful information [98]. Pattern recognition was successfully performed using ML-DSP (Machine Learning with Digital Signal Processing), a genomic classifier that achieves fast, accurate viral and bacterial classification at all taxonomic levels, which was developed by Randhawa et al. in order to improve the accuracy and speed of NGS [99]. Notably, it managed to classify 4710 bacterial genomes into phyla with an accuracy of 95.5%. ML-DSP was substantially faster than alignment-based classifiers (0.2 s vs. 134 s) and showed comparable classification accuracies when used on small datasets and superior accuracies for larger datasets. At present, artificial intelligence technologies are being evaluated for their application in the prevention, diagnosis and treatment of PJI [100].

When evaluating diagnostic biomarkers, although the accuracy of the used tests should be sufficiently high to identify PJI, the investigators should also consider other attributes of the test. First, two major concerns are the time elapsed from acquisition of the probes to the results and the moment of sampling. Considering that the mode of treatment for aseptic failure is completely different from that for the septic failure of implants, preoperative or intraoperative diagnosis is of utmost importance for a successful outcome. Synovial biomarkers have shown promising results in terms of ease of sampling, costs and speed; for example, the leukocyte esterase test can be performed in 5 min [101]. While providing excellent accuracy, other tests such as bacterial cultures inoculated with the sonication fluid of the explanted prosthesis or immunohistochemical analysis of the periprosthetic tissue can be utilized for PJI diagnosis only after surgery, thus losing the ability to influence intraoperative treatment decisions. A gold standard test should thoroughly diagnose PJI preoperatively, or be sufficiently fast enough to be performed intraoperatively such that the surgeons can act accordingly and increase the success rate of the treatment. Another major factor is cost. Compared to the leukocyte esterase test, the cost of the alpha-defensin immunoassay is substantially higher while providing similar accuracy and speed. Advanced molecular techniques such as PCR and mNGS are starting to grow in availability, and we expect the costs of tests involving such technologies to become more affordable in the near future.

## 7. Conclusions

Periprosthetic infection is a major complication after joint arthroplasty surgery that has been the subject of research worldwide due to the complexity of its treatment, long hospital stays, poor functional outcomes and high morbidity in patients, leading to high financial burdens on healthcare systems. One of the most challenging aspects of PJI is the difficulty of diagnosing the condition. Although the success of treatment depends mostly on the availability of a fast and accurate preoperative diagnostic, the clinical and paraclinical heterogeneity of PJI makes it almost impossible to currently develop a gold standard test for the disease. Multi-criteria diagnostic protocols are necessary in order to accurately detect such infections. We consider that the relevant field of research is heading in the right direction, with more serum, synovial and tissue biomarkers constantly being developed. In conjunction with novel molecular detection techniques, these biomarkers provide the best chance to achieve high diagnostic accuracy for PJI. Future studies focused on the most accurate biomarkers described in this study, including synovial antimicrobial peptides, synovial lipocalin-2 and calprotectin, are essential in order to improve the cost, simplicity, reproducibility and reliability of the tests.

## Figures and Tables

**Table 1:** PJI diagnosis via ICM, EBJIS and IDSA protocols.

PJI Definition				
ICM 2018 [8] (Probability of infection assessed by scoring system)			EBJIS 2021 [7] (Infection confirmed if one criterion is met)	IDSA 2013 [6] (Infection confirmed if one criterion is met)
Major criteria (one criterion shows infection)	Two positive cultures of the same organism		Sinus tract with evidence of communication to the joint or visualization of the prosthesis	The presence of a sinus tract that communicates with the prosthesis
	Sinus tract with evidence of communication to the joint or visualization of the prosthesis			
Preoperative score ≥6 Infected 2–5 Possibly Infected 0–1 Not infected	Elevated CRP or D-Dimer in Serum	2		
	Elevated Serum ESR	1		Acute inflammation on histopathologic examination of prosthetic tissue
	Elevated Synovial WBC or LE(++)	3	>3000 leukocytes/µL or >80% PMNs in synovial fluid	
	Positive Synovial Alpha-defensin	3		
	Elevated Synovial PMN%	2	Positive immunoassay or lateral-flow test for alpha-defensin from synovial fluid	Presence of purulence around the prosthesis without another known etiology
	Elevated Synovial CRP	1		
Intraoperative score ≥6 Infected 4–5 Inconclusive ≤3 Not infected	Preoperative Score	-	≥2 positive samples with the same microorganisms from intraoperative fluid or tissue or >50 CFU/mL of any organism from sonication fluid	
	Positive Histology	3		Two or more intraoperative cultures or combination of preoperative aspiration and intraoperative cultures that yield the same organism
	Positive Purulence	3	Presence of ≥5 neutrophils in ≥5 HPF (400× magnification) or presence of visible bacteria	
	Positive Single Culture	2		

**Table 2:** The sensitivities and specificities of the current diagnostic criteria for PJI.

Article	Article Type	Diagnostic Criterion	Sensitivity	Specificity
Saleh A et al. (2018) [14]	Review	Serum CRP	74–94%	20–100%
Saleh A et al. (2018) [14]	Review	Serum ESR	42–94%	33–87%
E. Ghanem et al. (2009) [19]	Cohort study (479 patients)	Serum CRP	91.1%	76.6%
E. Ghanem et al. (2009) [19]	Cohort study (479 patients)	Serum ESR	94.3%	70.2%
Berbari E. et al. (2010) [22]	Meta-analysis (3 studies)	Serum IL-6	97%	91%
Kai Xie et al. (2017) [23]	Meta-analysis (17 studies)	Serum IL-6	72%	89%
Sun X et al. (2024) [26]	Meta-analysis (9 studies)	Serum procalcitonin	44.1%	85.2%
Wang R. et al. (2022) [30]	Meta-analysis (10 studies)	Serum D-dimers	81%	74%
Worthington T. et al. (2010) [32]	Case–control study (46 patients)	Serum sICAM-1	94%	74%
Parvizi J. et al. (2012) [35]	Cohort study (59 samples)	Synovial CRP	84%	97.1%
Colin M Baker et al. (2022) [36]	Cohort study (621 patients)	Synovial CRP	74.2%	98%
Bonanzinga T. et al. (2016) [39]	Cohort study (156 patients)	Synovial alpha-defensin (immunoassay test)	97%	97%
Sufian S. Ahmad et al. (2018) [40]	Meta-analysis (42 studies)	Synovial alpha-defensin (immunoassay test)	80%	89%
Sufian S. Ahmad et al. (2018) [40]	Meta-analysis (42 studies)	Synovial alpha-defensin (ELISA test)	97%	97%
Chen Y et al. (2019) [41]	Meta-analysis (28 studies)	Synovial alpha-defensin (immunoassay test)	85%	96%
Chen Y et al. (2019) [41]	Meta-analysis (28 studies)	Synovial alpha-defensin (ELISA test)	92%	99%
Deirmengian et al. (2014) [33]	Cohort study (95 patients)	Synovial lipocalin-2	100%	100%
Vergara A et al. (2019) [44]	Cohort study (72 patients)	Synovial lipocalin-2	86.3%	77.2%
Huang Z et al. (2022) [45]	Cohort study (78 patients)	Synovial lipocalin-2	92%	98%
J. Parvizi et al. (2011) [58]	Cohort study (108 patients)	Synovial leukocyte esterase	80.6%	100%
Li J. et al. (2022) [51]	Meta-analysis (30 studies)	Synovial IL-6	87%	90%
Warren J. et al. (2021) [55]	Cohort study (123 patients)	Synovial calprotectin	98.1%	95.7%
Peng X. et al. (2022) [57]	Meta-analysis (7 studies)	Synovial calprotectin	94%	93%
Morawietz et al. (2009) [66]	Cohort study (147 samples)	CD15 marker—histopathology	73%	95%
Banke et al. (2020) [69]	Case–control study (25 patients)	HBD-3 marker—histopathology	100%	100%
Banke et al. (2020) [69]	Case–control study (25 patients)	LL-37 marker—histopathology	100%	100%
Cipriano C. et al. (2014) [72]	Cohort study (59 patients)	TLR-1 marker—histopathology	95%	100%
Watanabe S. et al. (2024) [77]	Meta-analysis (32 studies)	Preoperative fluid culture	63%	96%
Watanabe S. et al. (2024) [77]	Meta-analysis (32 studies)	Intraoperative tissue culture	71%	92%
Watanabe S. et al. (2024) [77]	Meta-analysis (32 studies)	Sonication fluid culture	78%	91%
Qu X. et al. (2013) [81]	Meta-analysis (14 studies)	16S rRNA—PCR	86%	91%
Tang Y et al. (2021) [82]	Meta-analysis (9 studies)	mNGS	63–96%	73–100%
Beguiristain I et al. (2023) [85]	Cohort study (107 patients)	Direct MALDI-TOF/MS	69%	94%
Kuo FC et al. (2020) [86]	Cohort study (77 patients)	Direct MALDI-TOF/MS	80%	-
Morgenstern C. et al. (2020) [90]	Cohort study (107 patients)	Microcalorimetry	39%	98%
Cichos KH et al. (2023) [91]	Cohort study (152 patients, 592 samples)	Microcalorimetry	83%	100%
